# New Molecular Targets and Lifestyle Interventions to Delay Aging Sarcopenia

**DOI:** 10.3389/fnagi.2014.00156

**Published:** 2014-07-02

**Authors:** Fabian Sanchis-Gomar, Helios Pareja-Galeano, Sara Mayero, Carme Perez-Quilis, Alejandro Lucia

**Affiliations:** ^1^Department of Physiology, Faculty of Medicine, University of Valencia and Fundación Investigación Hospital Clínico Universitario INCLIVA, Valencia, Spain; ^2^Department of Molecular and Mitochondrial Medicine, University Research Institute “Dr. Viña Giner”, Catholic University of Valencia “San Vicente Mártir”, Valencia, Spain; ^3^Department of Psychiatry, Hospital General Universitario, Valencia, Spain; ^4^Universidad Europea and Research Institute of Hospital 12 de Octubre (i+12), Madrid, Spain

**Keywords:** muscle atrophy, senescence factors, signaling pathways, frailty, pharmaceutical targets

The term sarcopenia was originally created to refer age-related loss of muscle mass with consequent loss of strength (Morley et al., 2001). There are now four international definitions of sarcopenia (Cruz-Jentoft et al., 2010; Muscaritoli et al., 2010; Morley et al., 2011). In essence they all agree, requiring a measure of walking capability [either low gait speed or a limited endurance (distance) in a 6-min walk], together with an appendicular lean mass of <2 SDs of a sex and ethnically corrected normal level for individuals 20–30 years old. Sarcopenia is a prevalent health problem among the elderly. On average, 5–13 and 11–50% of people aged 60−70 years and ≥80 years, respectively suffer sarcopenia with higher prevalences (68%) been reported in nursing home residents ≥70 years (Landi et al., 2012).

Sarcopenia needs to be differentiated from cachexia, which is a combination of both muscle and fat loss and is usually attributable to an excess of catabolic cytokines associated with a disease process (Argiles et al., 2010). Sarcopenia is a prime component of the frailty syndrome, and both sarcopenia and frailty are associated with increased disability, falls, hospitalization, nursing home admission, and mortality (Cesari and Vellas, 2012; Landi et al., 2012).

Medical efforts to develop treatments aiming at preventing aging sarcopenia as well as acute muscle atrophy and frailty in critical patients are considered a step forward in public health. Several hormonal therapies have been proposed for this purpose, such as those based on human growth hormone (hGH), IGF-1, testosterone, and stanozolol. However, the secondary effects associated with these therapies make it necessary to find novel non-toxic and non-hormonal therapies. In this way, elderly or bedridden patients may improve muscle function and decrease the degree of dependence associated with these populations. New drugs such as allopurinol or losartan (Sanchis-Gomar et al., 2011), all of them approved by the Food and Drugs Administration (FDA) and actually prescribed for the treatment of other diseases, could be useful in preventing loss of muscle mass in the described susceptible populations yet new pharmacological targets are needed.

## Novel Pharmacological Targets to Prevent Sarcopenia: Emerging Pathways to be Explored

### p16INK4a, NAD^+^, and sestrins pathways

In a recent manuscript, we proposed new targets for combating aging-related chronic illness (Pareja-Galeano et al., 2014). An altered mitochondrial homeostasis through reduced sirtuin 1 (SIRT1) activity induced by low nicotinamide adenine dinucleotide (NAD^+^) levels has been recently advocated as a hallmark of muscle aging. A depleted NAD^+^ pool could be the result of both the diminished NAD^+^ synthesis and increased NAD^+^ consumption that occurs with age (Gomes et al., 2014). Treatment of mice with NMN (an NAD^+^ precursor) can restore NAD^+^ levels and markers of mitochondrial function that decay with age, reversing muscle mitochondrial senescence (Prolla and Denu, 2014).

Another novel potential biomarker arising from recent animal research is the p16INK4a tumor suppressor. In geriatric mice, satellite cells lose their quiescent state owing to deregulation of p16INK4a, whereas repressing p16INK4a restores muscle regenerative capacity (Sousa-Victor et al., 2014). It is also known that p16INK4a expression increases with age, and its greater expression has been linked to increased attrition (Tsygankov et al., 2009). Recent evidence suggests that p16INK4a mRNA expression in peripheral blood T-lymphocytes is upregulated by gerontogenic behaviors such as tobacco use and physical inactivity, pointing to a critical role in age-related diseases (Song et al., 2010).

Sestrins are a third recently discovered hallmark of aging sarcopenia. Mammalian cells express sestrins (Sesn1, Sesn2, and Sesn3) in response to stress including DNA damage, oxidative stress, and hypoxia. Sestrins can inhibit the activity of the mammalian target of rapamycin complex 1 (mTORC1) through activation of AMP-dependent protein kinase (AMPK) (Lee et al., 2013). Sestrins prevent sarcopenia, insulin resistance, diabetes, and obesity. They also extend life and health span through activation of AMPK, suppression of mTORC1, and stimulation of autophagic signaling (Lee et al., 2013). We also proposed a possible role of the AMPK-modulating functions of sestrins in the benefits produced by exercise in older subjects (Sanchis-Gomar, 2013a).

### FGF21 and irisin: Potential therapeutic PGC-1α-related targets for aging and age-associated diseases

Circulating body levels of irisin and fibroblast growth factor 21 (FGF21) increase after cold exposure (Lee et al., 2014). Exercise-induced irisin secretion by working skeletal muscles, which could have evolved from shivering-related muscle contraction, might be a potential target of therapies designed to optimize weight control and metabolic profile (Lee et al., 2014). Hence, targeting irisin and FGF21, and particularly the key signaling molecule responsible for their secretion, the peroxisome proliferator-activated receptor gamma coactivator-1 α (PGC-1α), could identify new candidates to be included in the anti-aging armamentarium (Sanchis-Gomar, 2013b).

Irisin is an 112-amino acid glycoprotein, derived from the cleavage in working muscles – and subsequent secretion to the circulation – of a PGC-1α-dependent type I membrane protein, the fibronectin type III domain-containing protein 5 (FNDC5, 209 amino acids) (Bostrom et al., 2012). Exercise-released irisin might act as a hormone either locally within the muscle or targeting distant organs, particularly white adipose tissue, and increase total energy expenditure (Bostrom et al., 2012). Irisin production increases with chronic endurance exercise in mice and humans, and has been described to mitigate obesity and diet-induced insulin resistance (Bostrom et al., 2012), yet its levels decline with age (Sanchis-Gomar and Perez-Quilis, 2014). To explain exercise benefits on insulin resistance, we recently proposed the following pathway starting in muscle and targeting pancreatic β-cells: exercise-induced reactive oxygen species (ROS) → p38 → MAPK → PGC-1α → irisin → betatrophin → β-cell regeneration (Sanchis-Gomar and Perez-Quilis, 2014). This pathway could also be affected by aging. Interestingly, it has been also recently reported that disease-free centenarians have increased serum irisin levels (Emanuele et al., 2014). Exercise induces the expression of another PGC-1α – related hormone, FGF21 (Kim et al., 2013). Fasting drives the production of FGF21 in the liver, where it induces PGC-1α expression, thereby stimulating fatty acid oxidation, tricarboxylic acid cycle flux, and gluconeogenesis. In effect, mice lacking FGF21 are unable to fully induce PGC-1α expression in response to a prolonged fast and show impaired gluconeogenesis and ketogenesis (Potthoff et al., 2009). Thus, FGF21 plays an important role in ensuring metabolic regulation during progression from fasting to starvation.

**Figure 1:**
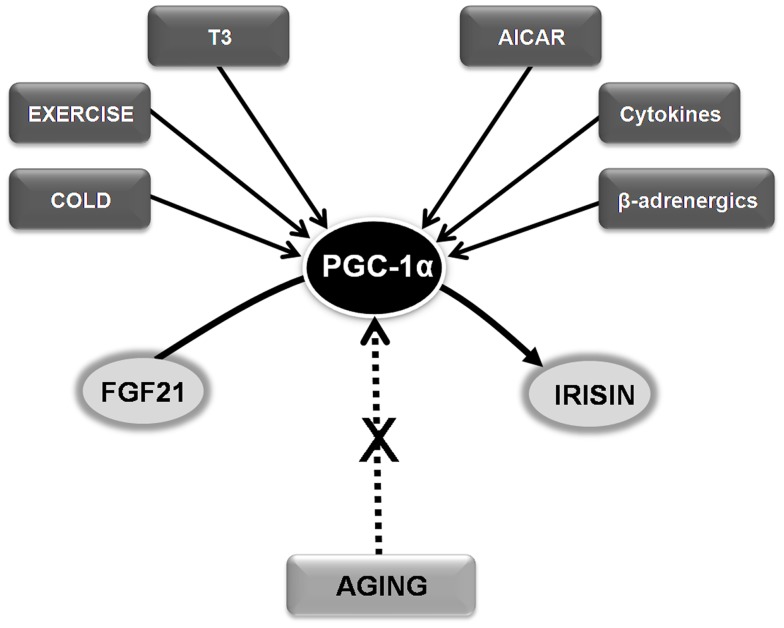
**Hypothesizing the role of FGF21-PGC-1α-Irisin axis in age-related conditions and sarcopenia**. See text for abbreviations.

Besides metabolic deregulation and increased insulin resistance, another important consequence of the aging process, reduced mitochondrial biogenesis, is also linked to abnormal PGC-1α signaling (Sanchis-Gomar and Derbre, 2014). Importantly, an age-related lack of muscle mitochondrial biogenesis can contribute to sarcopenia. PGC-1α knock-out mice and aged rats show a strikingly similar muscle phenotype: they are unable to express PGC-1α in response to the stimuli [i.e., exercise training, cold induction, or thyroid hormone (triiodothyronine – T3 – treatment)] that naturally up-regulate this molecule in young healthy rats (Derbre et al., 2012). Thus, maintaining normal PGC-1α responsiveness might help prevent age-related lack of muscle mitochondrial biogenesis (Derbre et al., 2012). In fact, several PGC-1α activators such as T3, cold induction, 5′-aminoimidazole-4-carboxamide-1-beta-d-ribofuranoside (AICAR), β-adrenergics, cytokines, and exercise have been postulated to prevent aging sarcopenia (Figure 1). The pioneer results by Lee et al. (2014) also suggest that targeting PGC-1α, e.g., using endocrine activators of brown fat function such as irisin and FGF21, might benefit the treatment of other age-related conditions, particularly metabolic diseases.

### Free-radical theory of aging questioned: Other molecular targets to prevent sarcopenia are needed

Treatments for age-related and disease-related muscle loss might improve active life expectancy in older people, and lead to substantial health-care savings and improved quality of life (Rastogi-Kalyani et al., 2014). However, the results of recent epidemiological studies (Perez et al., 2009) suggest that antioxidant supplementation does not lower the incidence of major age-associated diseases and might even increase the risk of death in some cases, have questioned the classic free-radical theory of aging (Gladyshev, 2014; Sanchis-Gomar et al., 2014). In fact, evidence mounts that ROS are important mediators of the health-promoting, life-span-extending capacity of regular exercise, as they play an important signaling role in a multitude of pathways including: angiogenesis, vascular distensibility, and up-regulation of PGC-1α, PGC-1α/nuclear respiratory factor 1-stimulated mitochondrial biogenesis or cytoprotective “stress proteins” (heme oxygenase 1, heat shock proteins like HSP60 and HSP70) in muscle (Fiuza-Luces et al., 2013; Sanchis-Gomar and Derbre, 2014). This means that antioxidant interventions are unlikely to help combat sarcopenia. Moreover, anti-ROS strategies could even aggravate sarcopenia. Thus, a major switch in strategy is proposed and investigators are now focusing on myostatin and follistatin as promising molecular targets of anti-sarcopenia treatments. Myostatin is a skeletal muscle-specific secreted peptide, pertaining to the transforming growth factor-β (TGF-β) family member, that inhibits myoblast proliferation and consequently muscle mass/strength by acting as a negative regulator of mTOR-signaling (Garatachea et al., 2013). Mice treated with losartan, an angiotensin II receptor antagonist, were protected against loss of muscle mass and this effect was mediated by activation of the IGF-1/Akt/mTOR pathway (Sanchis-Gomar et al., 2011). These observations highlight the importance of IGF-1/GH balance in longevity and may be of therapeutic interest when targeting the undesirable effects of aging, especially at the muscle level (Sandri et al., 2013).

Myostatin inhibition by agents capable of blocking the myostatin signaling pathway such as ACVR2B (a soluble form of the activin type IIB receptor) could have important applications in the treatment of human muscle degenerative diseases (Lee et al., 2005). In addition, the growth and derived factor (GDF)-associated serum proteins-1 (GASP-1) and 2 (GASP-2), which show competitive binding with proteins capable of inhibiting myostatin, decrease muscle weight and impair muscle regeneration ability in mice (Lee and Lee, 2013). Moreover, the inhibition of the myostatin/activin A signaling pathway is sufficient to induce muscle hypertrophy and can be an effective therapeutic approach for increasing muscle growth in disease settings characterized by satellite cell dysfunction. Finally, the propeptide follistatin, a myostatin antagonist, might be a useful agent for enhancing muscle growth in human therapeutic applications. In fact, increasing follistatin circulating concentrations might help prevent and treat frailty, as well as the cardiometabolic complications associated with androgen-deprivation therapy (Sanchis-Gomar, 2013b).

## Importance of Lifestyle Interventions to Delay Sarcopenia

Another important tool in the prevention of sarcopenia is physical exercise (some of the molecular pathways involved have been discussed above). Particularly, exercise training programs with resistance (strength) exercises (i.e., movements performed against a specific external force that is regularly increased during training) are especially useful for improving muscle mass or strength in the elderly (Liu and Latham, 2009), including in the oldest-old (people aged 90 years or over) (Fiatarone et al., 1990).

On the other hand, autophagy also plays an important key role both in the modulation of lifespan and sarcopenia (Madeo et al., 2010; Schiavi et al., 2013). Interestingly, autophagy is required to maintain muscle mass and thus to prevent sarcopenia (Masiero et al., 2009; Neel et al., 2013). In effect, failure of autophagy contributes to the sarcopenic phenotype observed in premature aging (Joseph et al., 2013). For this reason, physical exercise and calorie restriction are commonly recommended to prevent sarcopenia since both of them modulate autophagy signaling (Marzetti et al., 2008; Wohlgemuth et al., 2010).

## Final Opinion

As an essential step for the prevention of aging-related diseases, and specifically, sarcopenia, more basic research is needed on the main cellular hallmarks of muscle senescence. There is a plethora of potential molecular signals that are candidates to be targeted in future treatment strategies aiming at combating sarcopenia, a devastating effect of aging that is often overlooked.

## Conflict of Interest Statement

The authors declare that the research was conducted in the absence of any commercial or financial relationships that could be construed as a potential conflict of interest.
